# Association of residual lipoprotein-cholesterol and atherogenic index of plasma with in-hospital major adverse cardiovascular and cerebrovascular events after percutaneous coronary intervention in patients with acute ST-segment elevation myocardial infarction

**DOI:** 10.1186/s12872-026-05555-9

**Published:** 2026-01-26

**Authors:** Ni An, Hailong Lu, Tian Liu, Defeng Pan

**Affiliations:** 1 Department of Geriatrics, The Affiliated Hospital of Xuzhou Medical University, Xuzhou, 221000 China; 2 Department of Cardiology, The Affiliated Hospital of Xuzhou Medical University, Xuzhou, 221000 China

**Keywords:** ST-segment elevation myocardial infarction, Residual lipoprotein-cholesterol, Atherogenic index of plasma, Major adverse cardiovascular and cerebrovascular vascular events

## Abstract

**Background:**

In recent years, non-traditional lipid indices have emerged as significant predictors for cardiovascular events following emergency percutaneous coronary intervention (PCI) for ST-segment elevation myocardial infarction (STEMI). However, the relationship of residual lipoprotein-cholesterol (RLP-C) and atherogenic index of plasma (AIP) with in-hospital outcomes, especially their predictive value for major adverse cardiovascular and cerebrovascular events (MACCEs) after PCI in STEMI patients, remains underexplored and warrants further investigation.

**Methods:**

This retrospective cohort study included 526 STEMI patients who underwent emergency PCI between January 2023 and August 2024. We collected baseline demographic, clinical, and laboratory data. RLP-C and AIP were calculated from lipid profiles obtained before PCI. Independent predictors of in-hospital MACCEs were identified using multivariate logistic regression, and model discrimination was evaluated using receiver operating characteristic (ROC) curve analysis.

**Results:**

Among 526 STEMI patients receiving PCI, 92 (17.49%) developed in-hospital MACCEs. Multivariate analysis identified RLP-C (OR = 3.97, 95%CI: 1.71–9.21; *P* = 0.001) and AIP (OR = 2.42, 95%CI: 1.01–5.76; *P* = 0.047) as independent predictors of MACCEs after adjusting for conventional risk factors. The integrated model with hsTnT, ApoB, RLP-C, and AIP demonstrated superior predictive accuracy (AUC 0.744). Dose-response analysis revealed a borderline nonlinear relationship between AIP and MACCEs risk (P for nonlinearity = 0.050), while RLP-C demonstrated a linear dose-response relationship with MACCEs risk (P for nonlinearity = 0.522).

**Conclusion:**

RLP-C and AIP are independent predictors of in-hospital MACCEs following PCI in STEMI patients. Combined assessment of these indices improves risk stratification and may facilitate early targeted interventions to improve outcomes.

**Supplementary Information:**

The online version contains supplementary material available at 10.1186/s12872-026-05555-9.

## The advantages and limitations of this paper

### Advantages

This study provides novel evidence on the predictive value of RLP-C and AIP for in-hospital MACCEs in STEMI patients undergoing PCI. Our findings demonstrate that: First, RLP-C and AIP were independently associated with in-hospital MACCEs in STEMI patients. Second, incorporating these into risk models significantly improved predictive accuracy for MACCEs compared to conventional risk factors alone. Third, combined assessment of RLP-C and AIP identified high-risk patient subgroups with substantially higher event rates. These findings suggest that RLP-C and AIP could serve as practical biomarkers for early risk stratification in clinical practice.

### Limitations

Our study has several limitations. First, as a single-center retrospective analysis limited to in-hospital outcomes, our findings may have limited generalizability and require external validation. Second, while we adjusted for numerous confounders in multivariable analysis, residual confounding from unmeasured variables (such as detailed anticoagulant regimens, specific dietary patterns, and physical activity levels) remains possible. Third, RLP-C and AIP were measured during the acute phase of STEMI, when various physiological disturbances may act as residual confounders affecting lipid levels independent of baseline status. While this represents a methodological limitation, it also reflects clinical reality where risk stratification must occur rapidly at presentation. The consistent independent predictive value of these markers across multivariable analyses, despite potential acute-phase influences, suggests they capture meaningful pathophysiological processes relevant to short-term outcomes. Fourth, our final multivariable model included 11 predictors with 92 outcome events, which may raise concerns about model overfitting. Although we employed strict forward selection criteria and assessed multicollinearity using variance inflation factors, and confirmed good model fit using the Hosmer-Lemeshow test, external validation remains essential to confirm the stability of our findings. Fifth, we observed an association between infarct-related artery distribution and outcomes, particularly a higher proportion of RCA lesions among patients with MACCEs, which may reflect specific pathophysiological mechanisms requiring further investigation. Finally, while our sample size provided adequate power for primary analysis, prospective multicenter validation is warranted before clinical implementation of these lipid indices.

## Introduction

Cardiovascular disease (CVD), particularly acute myocardial infarction (AMI), remains a major public health burden and a leading cause of mortality worldwide [1]. This imposes substantial economic burdens on healthcare systems globally, affecting both China and other nations. The introduction of guideline-based medication and percutaneous coronary intervention (PCI) procedures has considerably improved the prognosis of acute ST-segment elevation myocardial infarction (STEMI) [2]. Despite standardized treatment protocols during hospitalization, STEMI patients who received PCI remain at significant risk for major adverse cardiovascular and cerebrovascular events (MACCEs). This persistent risk underscores the need for improved risk prediction tools and more effective management strategies. Traditional risk factors including advanced age, smoking, hypertension, diabetes mellitus, hyperuricemia, hyperlipidemia, and triglyceride glucose-body mass index (TyG-BMI) have been recognized as established risk factors for CVD [3, 4]. In recent years, more non-traditional lipid indices such as residual lipoprotein-cholesterol (RLP-C), atherogenic index of plasma (AIP), and small and dense low-density lipoprotein-cholesterol (sdLDL-C) have been found to be closely related to atherosclerosis [5, 6].

While the American Heart Association has classified RLP-C as a risk factor for atherosclerosis in general populations [7], and studies have demonstrated AIP’s predictive value for cardiovascular events in broader cohorts [8–10], limited evidence exists specifically for STEMI patients who received PCI. The relationship between these non-traditional lipid markers and in-hospital MACCEs in this specific patient population remains insufficiently characterized. Therefore, this retrospective study specifically focused on STEMI patients who received PCI to evaluate the predictive role of RLP-C and AIP for in-hospital MACCEs. By examining the relationship between these lipid markers and adverse outcomes in this high-risk population, we aim to identify novel biomarkers that can enhance risk stratification strategies and ultimately improve clinical outcomes for STEMI patients undergoing PCI.

## Materials and methods

### Study population

**Fig. 1:**
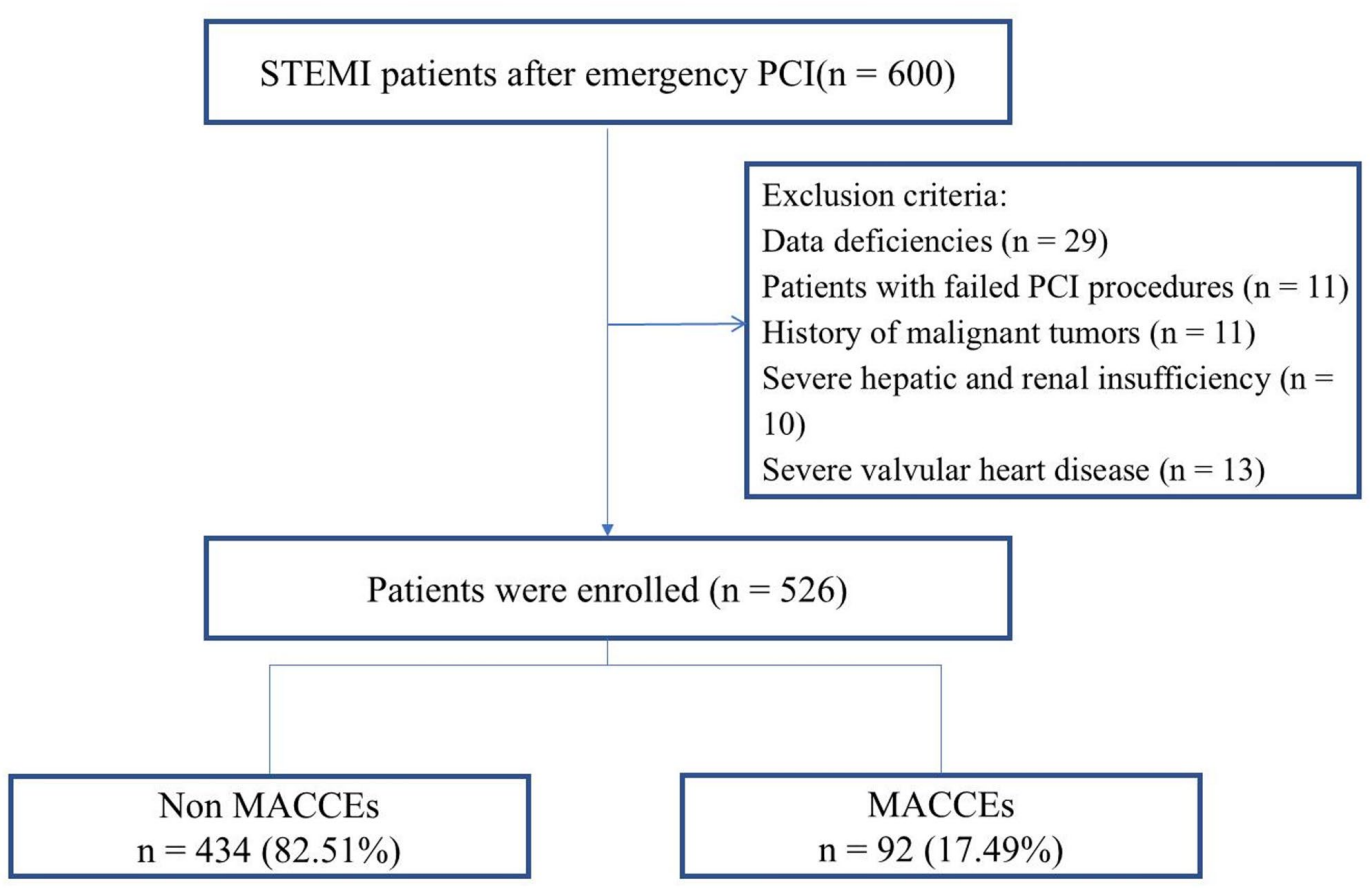
The flowchart of study

Consecutive STEMI patients who received emergency PCI at the Affiliated Hospital of Xuzhou Medical University between January 2023 and August 2024 were enrolled. Inclusion criteria: (1) age 18–80 years; (2) STEMI identified using the third edition of the global definition of myocardial infarction [11]; (3) successful completion of PCI within 24 h of symptom onset; (4) continuous ECG monitoring in the hospital. Exclusion criteria: (1) malignant tumors, immune system diseases; (2) severe hepatic and renal insufficiency; (3) previous history of coronary artery bypass grafting, coronary intervention, and other organic heart diseases such as combined heart valve disease, rheumatic heart disease, and cardiomyopathy; (4) incomplete and missing clinical data. Initially, 600 patients were screened for eligibility, and after applying the exclusion criteria, 526 patients were finally enrolled. The study population was divided into two groups: those without MACCEs (*n* = 434) and those with MACCEs (*n* = 92) (Fig. 1). This retrospective study was approved by the Ethics Committee of The Affiliated Hospital of Xuzhou Medical University (approval number: XYFY2024-KL630-01).

### Data collection

Clinical and laboratory data were extracted from the hospital information system by trained researchers. Blood samples were collected in the emergency department immediately upon admission, prior to PCI procedure and administration of any lipid-lowering medications. The symptom onset-to-balloon time was systematically recorded for all patients. Data collection included five domains: (1) Demographics (age, gender, BMI); (2) Comorbidities (current smoking, diabetes, hypertension, prior stroke); (3) Clinical parameters (blood pressure, heart rate, LVEF, Killip class); (4) Angiographic characteristics (culprit vessels, TIMI flow grade); and (5) Laboratory measurements including complete blood count, renal and hepatic function tests, cardiac biomarkers (peak NT-proBNP, peak CK-MB), inflammatory markers (peak hsCRP), and comprehensive lipid profiles. Data on medication history included pre-admission statin use. In-hospital medications, encompassing statin therapy, antiplatelet drugs, Sacubitril Sodium Tablets, ACEI/ARB, β-blockers, and spironolactone, were also recorded. The following formulas were used to determine RLP-C and AIP: RLP-C = TC - HDL-C - LDL-C and AIP = log (TG/HDL-C) [12, 13].

### Outcomes

The observational outcome in this study was in-hospital MACCEs, defined as a composite of all-cause death, reinfarction, mechanical complications of infarction, cardiogenic shock, and ischemic stroke. The diagnostic criteria for ischemic stroke were based on the definition by the American Heart Association/American Stroke Association (AHA/ASA) [14]. Cardiogenic shock was documented only for patients who were not in shock at admission [15]. Reinfarction was defined as a new AMI occurring within 28 days of the index event, and mechanical complications of infarction included papillary muscle rupture, ventricular free wall rupture, and ventricular septal rupture occurring during the index hospitalization [16]. If a patient experienced numerous MACCEs throughout the hospitalization, only one was counted in the MACCEs calculation.

### Statistical methods

The data were analyzed using SPSS (version 27.0) and R (version 4.3.1). Normality was assessed using the Kolmogorov-Smirnov test. Continuous variables with normal distribution were expressed as mean ± standard deviation (x̄ ± s), and compared using the independent t-test between two groups. Non-normally distributed continuous variables were presented as median (interquartile range) [M (Q1, Q3)] and analyzed using the Mann-Whitney U test. Categorical variables were reported as counts and percentages, and between-group comparisons were performed using the χ² test.

The correlation between RLP-C and AIP was evaluated using Spearman’s correlation coefficient. STEMI patients who underwent PCI were stratified into four subgroups based on median values of AIP and RLP-C (low AIP-low RLP-C, low AIP-high RLP-C, high AIP-low RLP-C, and high AIP-high RLP-C). The stratification was validated by one-way ANOVA (both *P* < 0.001), and the incidence of in-hospital MACCEs was compared across these subgroups to evaluate their combined prognostic impact.

For multivariable logistic regression analysis, we employed a forward stepwise selection approach. Variables with *P* < 0.1 in univariate logistic regression analyses were initially considered as candidate variables. The forward selection process used an entry criterion of *P* < 0.05 and a removal criterion of *P* > 0.10. At each step, the variable with the smallest P-value was entered into the model if it met the entry criterion, and variables already in the model were removed if they exceeded the removal criterion. The final multivariate model included peak hsTnT, peak CK-MB, peak hs-CRP, TG, TC, HDL-C, ApoB, ALP, LVEF, AIP, and RLP-C. Multicollinearity was assessed using variance inflation factors (VIFs), with VIF > 5 indicating significant collinearity. Model fit was evaluated using the Hosmer-Lemeshow goodness-of-fit test. Results are presented as odds ratios (OR) with 95% confidence intervals (CI).

The predictive performance of RLP-C and AIP for in-hospital MACCEs was evaluated using receiver operating characteristic (ROC) curve analysis, with calculation of the area under the curve (AUC), optimal cutoff values, sensitivity, and specificity. The dose-response relationships between AIP/RLP-C and in-hospital MACCEs were assessed using restricted cubic splines (RCS) with three knots positioned at the 10th, 50th, and 90th percentiles of each biomarker’s distribution. Nonlinearity was formally tested using likelihood ratio tests comparing models with and without nonlinear terms. Statistical significance was defined as *P* < 0.05.

## Results

### Baseline characteristics

This study included 526 patients with STEMI who received PCI, with an in-hospital MACCEs incidence of 17.49% (92 cases). Baseline analysis revealed that patients who experienced MACCEs had significantly higher levels of RLP-C and AIP. They also had poorer cardiac function indicators (lower LVEF values, higher Killip class ≥ 2), longer onset-to-balloon time, lower post-PCI TIMI flow grade 3 rates, and elevated myocardial injury markers (hsTnT, CK-MB) and hsCRP (all *P* < 0.05). No significant differences were observed between groups in age, sex, traditional cardiovascular risk factors (hypertension, diabetes, smoking), or most medications. Notably, 389 patients (73.95%) were statin-naïve, and prior statin use was evenly distributed between groups (*P* = 0.291). This supports RLP-C and AIP as potential independent predictors of adverse outcomes after PCI (Table 1).

**Table 1:** Baseline data comparison between groups

Variables	Non MACCEs (*n* = 434)	MACCEs (*n* = 92)	*P*
Age, (years)	57.00 (50.00, 65.00)	58.00 (49.50, 68.00)	0.647
BMI, (kg/m^2^)	25.32 (23.44, 27.68)	25.95 (23.63, 27.95)	0.570
Systolic blood pressure, (mm/Hg)	127.00 (115.00, 141.00)	125.00 (110.75, 140.00)	0.329
Diastolic blood pressure, (mm/Hg)	80.00 (72.00, 89.00)	79.00 (70.00, 87.25)	0.575
Heart rate, (times/min)	78.00 (69.25, 87.00)	78.00 (70.75, 89.00)	0.526
Peak hsTnT, (ng/L)	2397.50 (965.80, 5136.75)	3738.50 (1903.00, 8552.50)	< 0.001*
Peak CK-MB, (ng/L)	116.15 (38.50, 263.12)	175.05 (76.28, 300.00)	0.021*
Peak NTproBNP, (pg/mL)	1070.00 (558.75, 1887.80)	1427.00 (545.00, 2640.25)	0.068
Peak hsCRP, (mg/L)	24.25 (10.53, 57.08)	37.30 (12.62, 71.22)	0.042*
TC, (mmol/L)	4.28 (3.62, 4.99)	4.50 (3.92, 5.51)	0.008*
TG, (mmol/L)	1.42 (0.97, 2.00)	1.87 (1.33, 3.08)	< 0.001*
LDL-C, (mmol/L)	2.76 (2.20, 3.38)	2.77 (2.17, 3.57)	0.496
HDL-C, (mmol/L)	0.92 (0.79, 1.06)	0.85 (0.75, 1.00)	0.003*
ApoB, (g/L)	0.90 (0.74, 1.07)	0.96 (0.80, 1.16)	0.006*
ApoA1, (g/L)	1.02 (0.90, 1.17)	1.02 (0.92, 1.21)	0.462
Lp(a), (mg/L)	225.00 (140.25, 343.25)	256.50 (160.75, 380.50)	0.083
eGFR, (mL/min/1.73 m^2^)	118.28 (100.38, 120.00)	115.53 (102.77, 120.00)	0.628
LVEF, (%)	53.00 (50.00, 57.00)	51.00 (48.75, 53.75)	< 0.001*
HbA1c, (%)	5.80 (5.60, 6.50)	5.95 (5.57, 6.93)	0.522
Glucose, (mg/dl)	5.66 (5.01, 6.95)	5.99 (5.27, 7.20)	0.125
WBC, (10^9/L)	9.90 (7.90, 11.90)	10.65 (8.70, 12.25)	0.072
NE, (10^9/L)	7.62 (5.95, 9.91)	8.76 (6.39, 10.29)	0.127
LYM, (10^9/L)	1.40 (1.00, 1.90)	1.30 (0.97, 1.70)	0.488
HGB, (g/L)	137.00 (126.00, 148.00)	137.00 (123.00, 146.25)	0.804
PLT, (10^9/L)	213.00 (181.00, 251.00)	209.50 (183.00, 246.00)	0.492
ALB, (umol/L)	38.00 (35.90, 40.88)	39.35 (35.40, 41.75)	0.107
UA, (U/L)	300.00 (244.50, 359.75)	310.00 (256.00, 391.50)	0.252
ALP, (U/L)	72.00 (61.25, 86.00)	79.50 (70.00, 91.00)	0.001*
RLP-C	0.55 (0.36, 0.74)	0.75 (0.50, 1.14)	< 0.001*
AIP	0.19 (-0.01, 0.37)	0.33 (0.16, 0.56)	< 0.001*
Male, n(%)	368 (84.79)	77 (83.70)	0.791
Smoking, n(%)	233 (53.69)	49 (53.26)	0.941
Hypertension, n(%)	198 (45.62)	42 (45.65)	0.996
Diabetes, n(%)	117 (26.96)	21 (22.83)	0.413
Stroke, n(%)	53 (12.21)	10 (10.87)	0.719
Killip class ≥ 2, n(%)	45 (10.37)	19 (20.65)	0.006*
IRA			0.052
IRA-LAD, n(%)	213 (49.08)	33 (35.87)	
IRA-LCX, n(%)	90 (20.74)	21 (22.83)	
IRA-RCA, n(%)	131 (30.18)	38 (41.30)	
Pre-TIMI ≤ 1, n(%)	331 (76.27)	77 (83.70)	0.121
Post-TIMI = 3, n(%)	430 (99.08)	86 (93.48)	0.002*
Onset-to-balloon time, (h)	7.00 (4.00, 12.00)	8.50 (5.00, 14.00)	0.022*
Statins, n(%)	425 (97.93)	90 (97.83)	1.000
Previous statins, n(%)	109 (25.12)	28 (30.43)	0.291
Sacubitril Sodium Tablets, n(%)	177 (40.78)	28 (30.43)	0.064
ACEI/ARB, n(%)	95 (21.89)	20 (21.74)	0.975
β-blockers, n(%)	372 (85.71)	80 (86.96)	0.756
Spironolactone, n(%)	29 (6.68)	13 (14.13)	0.017*
Antiplatelet drug, n(%)	431 (99.31)	92 (100.00)	1.000

*BMI* Body mass index, *LVEF* Left ventricular ejection fraction, *TC* Serum total cholesterol, *TG* Serum triglyceride, *LDL-C* Low-density lipoprotein cholesterol, *HDL-C* High-density lipoprotein cholesterol, *ApoB* Apolipoprotein B, *ApoA1* Apolipoprotein A-1, *Lp(a)* Lipoprotein(a), *hsTnT* High-sensitivity troponin T, *NT-proBNP* N-terminal pro B-type natriuretic peptide, *Peak CK-MB* Peak creatine kinase isoenzymes, *peak hsCRP* Peak high sensitivity c-reactive protein, *eGFR* Estimated glomerular filtration rate, *WBC* White blood cell, *NE* Neutrophils, *LYM* Lymphocyte, *HGB* Hemogloin, *PLT* Platelet, *ALB* Albumin, *UA* Uric acid, *ALP* Alkaline phosphatase, *RLP-C* Residual lipoprotein-cholesterol, *AIP* Atherogenic index of plasma, *LCX* Left circumflex branch, *LAD* Left anterior descending branch, *RCA* Right coronary artery, *TIMI* Thrombolysis in myocardial infarction, *ARB* Angiotensin II receptor antagonist, *ACEI* Angiotensin-converting enzyme inhibitors

### In-hospital outcomes

**Fig. 2:**
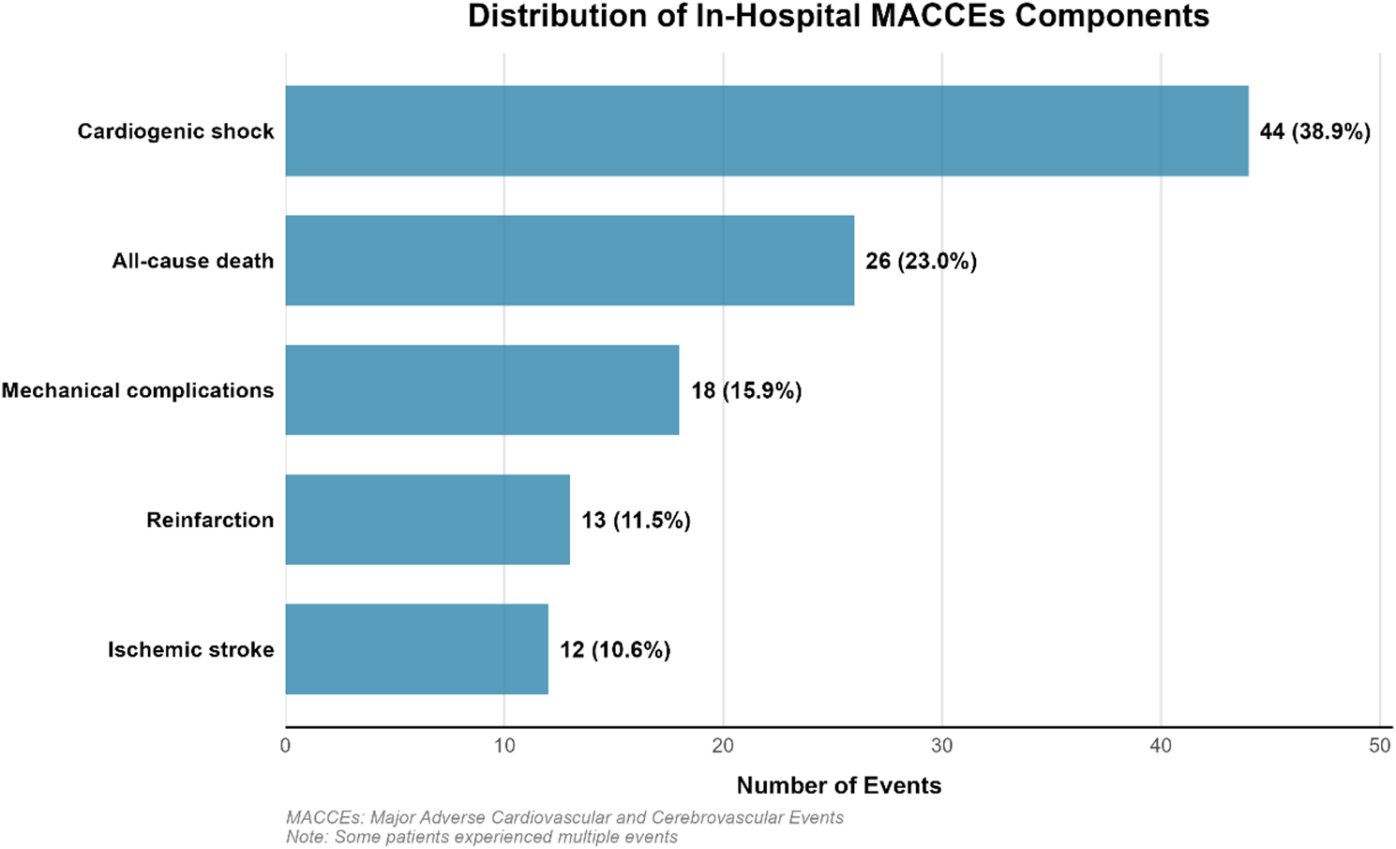
Distribution of individual components of in-hospital MACCEs

Among the 92 patients with STEMI who received PCI and experienced in-hospital MACCEs, cardiogenic shock was the most frequent component (44 events, 38.9%), followed by all-cause death (26 events, 23.0%), mechanical complications (18 events, 15.9%), reinfarction (13 events, 11.5%), and ischemic stroke (12 events, 10.6%). Stratification by combined AIP and RLP-C levels revealed a graded increase in adverse outcomes across the four groups. The highest rates of all-cause death (8.3%) and cardiogenic shock (12.0%) were observed in the group with both high AIP and high RLP-C, supporting the combined prognostic value of these two lipid indices in patients with STEMI who received PCI (Table 2; Fig. 2).

**Table 2:** In-Hospital outcomes stratified by the combined levels of AIP and RLP-C

In-hospital Outcomes	Low AIP - Low RLP-C (*n* = 190)	Low AIP - High RLP-C (*n* = 73)	High AIP - Low RLP-C (*n* = 71)	High AIP - High RLP-C (*n* = 192)
all-cause death	2 (1.1%)	3 (4.1%)	5 (7.0%)	16 (8.3%)
Mechanical complications	2 (1.1%)	3 (4.1%)	3 (4.2%)	10 (5.2%)
reinfarction	1 (0.5%)	4 (5.5%)	3 (4.2%)	5 (2.6%)
cardiogenic shock	9 (4.7%)	9 (12.3%)	3 (4.2%)	23 (12%)
Ischemic stroke	1 (0.5%)	1 (1.4%)	7 (9.9%)	3 (1.6%)

Patients were categorized into four groups based on median cutoffs of AIP and RLP-C. The grouping strategy was statistically validated by one-way ANOVA (both *P* < 0.001), confirming its efficacy in distinguishing patient risk tiers

### Associations of RLP-C and AIP with cardiovascular risk factors

Spearman correlation analysis revealed strong positive correlations between RLP-C and AIP with TG (*r* = 0.72 and *r* = 0.94, respectively) and moderate positive correlations with ApoB (*r* = 0.31 and *r* = 0.27, respectively). Both lipid indices demonstrated significant negative correlations with HDL-C (*r*=-0.24 and *r*=-0.62, respectively). RLP-C and AIP were highly correlated with each other (*r* = 0.67), reflecting their shared metabolic pathways.

**Fig. 3:**
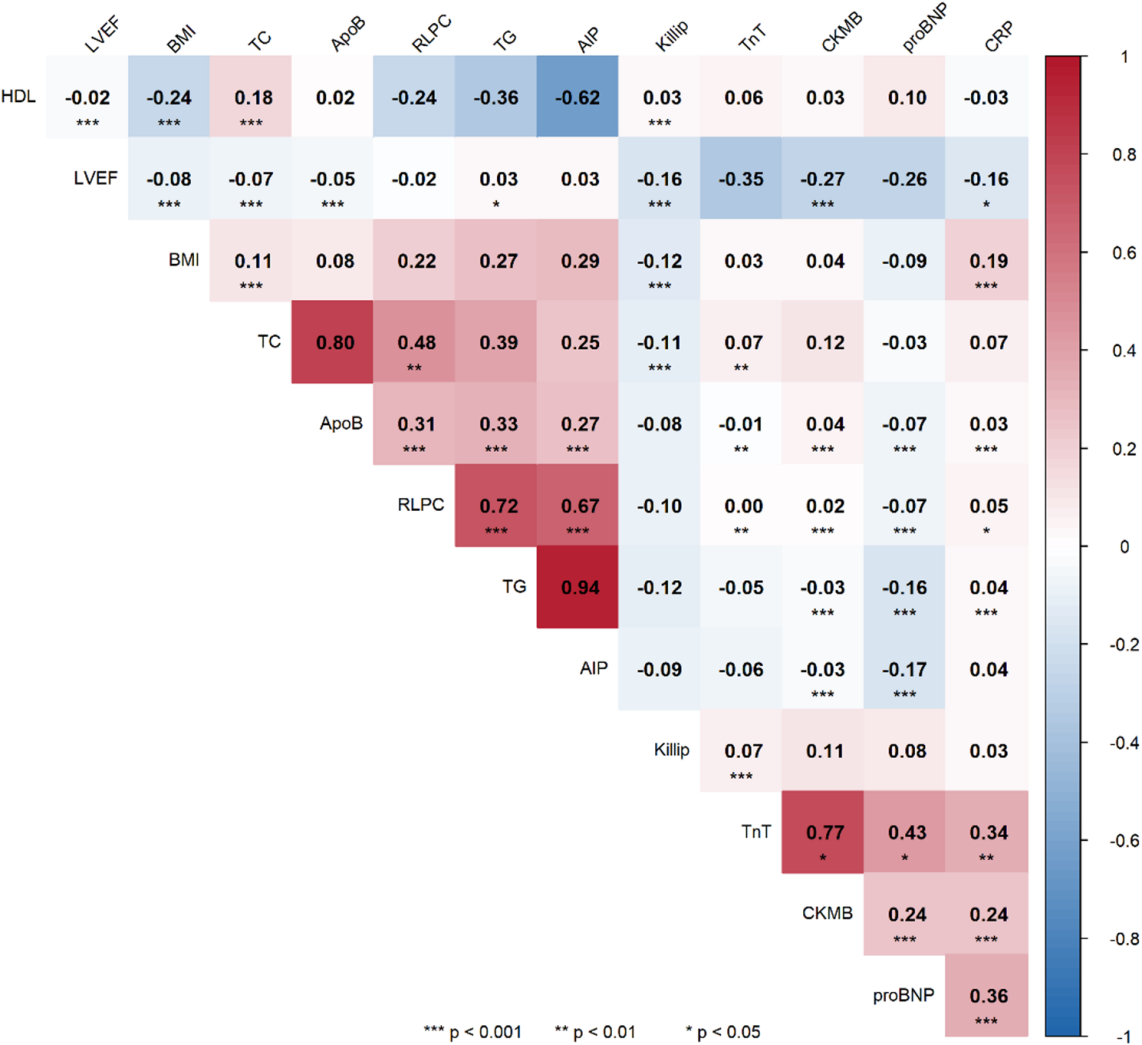
Correlation of RLP-C, AIP index and correlation with cardiovascular related factors. The heatmap displays correlation coefficients (r) between pairs of variables, with color intensity indicating the strength and direction of correlation. (blue: positive, red: negative). Asterisks denote statistical significance. (*P < 0.05, **P < 0.01, ***P < 0.001)

The lipid indices showed minimal correlation with myocardial injury markers. RLP-C had negligible correlation with hsTnT and weak correlation with CK-MB. Similarly, AIP showed weak correlations with both hsTnT and CK-MB. Both indices exhibited weak negative correlations with Killip class (*r*=-0.10 for both), indicating limited associations with hemodynamic status (Fig. 3).

### Logistic analysis

Following our pre-specified variable selection protocol, the multivariate logistic regression analysis identified RLP-C and AIP as independent predictors of in-hospital MACCEs. Elevated RLP-C levels significantly increased MACCEs risk (OR = 3.97, 95% CI: 1.71–9.21; *P* = 0.001) after adjusting for myocardial injury markers, inflammatory parameters, and conventional lipids. Higher AIP also independently predicted MACCEs (OR = 2.42, 95%CI: 1.01–5.76; *P* = 0.047). Additionally, hsTnT (OR = 1.01, 95%CI: 1.01–1.01; *P* = 0.027and ApoB (OR = 1.76 95%CI: 1.11–2.79; *P* = 0.016) remained significant predictors. Notably, traditional lipid parameters (TG, TC, HDL-C) were significant in univariate analysis but lost significance in the multivariate model, suggesting RLP-C and AIP provide prognostic value beyond conventional lipid markers. These findings support RLP-C and AIP as potential biomarkers for risk stratification in patients with STEMI who received PCI (Table 3).

**Table 3:** Logistic regression analysis

Variables	Univariate Logistic Regression Analysis		Multivariate Logistic Regression Analysis	
	*P*	OR (95%CI)	*P*	OR (95%CI)
Peak hsTnT, (ng/L)	< 0.001*	1.01 (1.01 ~ 1.01)	0.027*	1.01 (1.01 ~ 1.01)
Peak CK-MB, (ng/L)	0.020*	1.01 (1.01 ~ 1.01)	0.799	1.00 (1.00 ~ 1.00)
Peak hsCRP, (mg/L)	0.196	1.00 (1.00 ~ 1.01)	0.938	1.00 (0.99 ~ 1.01)
TG, (mmol/L)	< 0.001*	1.39 (1.20 ~ 1.62)	0.236	0.75 (0.47 ~ 1.20)
TC, (mmol/L)	0.002*	1.41 (1.14 ~ 1.76)	0.057	0.61 (0.36 ~ 1.01)
HDL-C, (mmol/L)	0.004*	0.18 (0.06 ~ 0.58)	0.317	3.02 (0.35 ~ 26.24)
ApoB, (g/L)	0.002*	1.44 (1.15 ~ 1.80)	0.016*	1.76 (1.11 ~ 2.79)
ALP, (U/L)	0.279	1.00 (1.00 ~ 1.01)	0.180	1.00 (1.00 ~ 1.01)
LVEF, (%)	0.003*	0.95 (0.92 ~ 0.98)	0.055	0.96 (0.92 ~ 1.00)
AIP	< 0.001*	2.02 (1.58 ~ 2.58)	0.047*	2.42 (1.01 ~ 5.76)
RLP-C	< 0.001*	4.86 (2.83 ~ 8.33)	0.001*	3.97 (1.71 ~ 9.21)

Variance inflation factors (VIFs) were calculated to assess multicollinearity, with VIF values as follows: hsTnT = 2.291, CK-MB = 2.173, hsCRP = 1.078, TC = 4.619, ALP = 1.009, LVEF = 1.141, AIP = 6.440, RLP-C = 2.031, ApoB = 3.513, HDL-C = 2.725, TG = 3.591

*hsTnT* High-sensitivity troponin T, *peak-hsCRP* Peak high sensitivity c-reactive protein, *peak CK-MB* Peak creatine kinase-MB, *TC* Serum total cholesterol, *TG* Serum triglyceride, *HDL-C* High-density lipoprotein cholesterol, *ApoB* Apolipoprotein B, *LVEF* Left ventricular ejection fraction, *RLP-C* Residual lipoprotein-cholesterol, *AIP* Atherogenic index of plasma (standardized using z-score transformation in multivariate analysis)

### Predictive value for in-hospital MACCEs

**Fig. 4:**
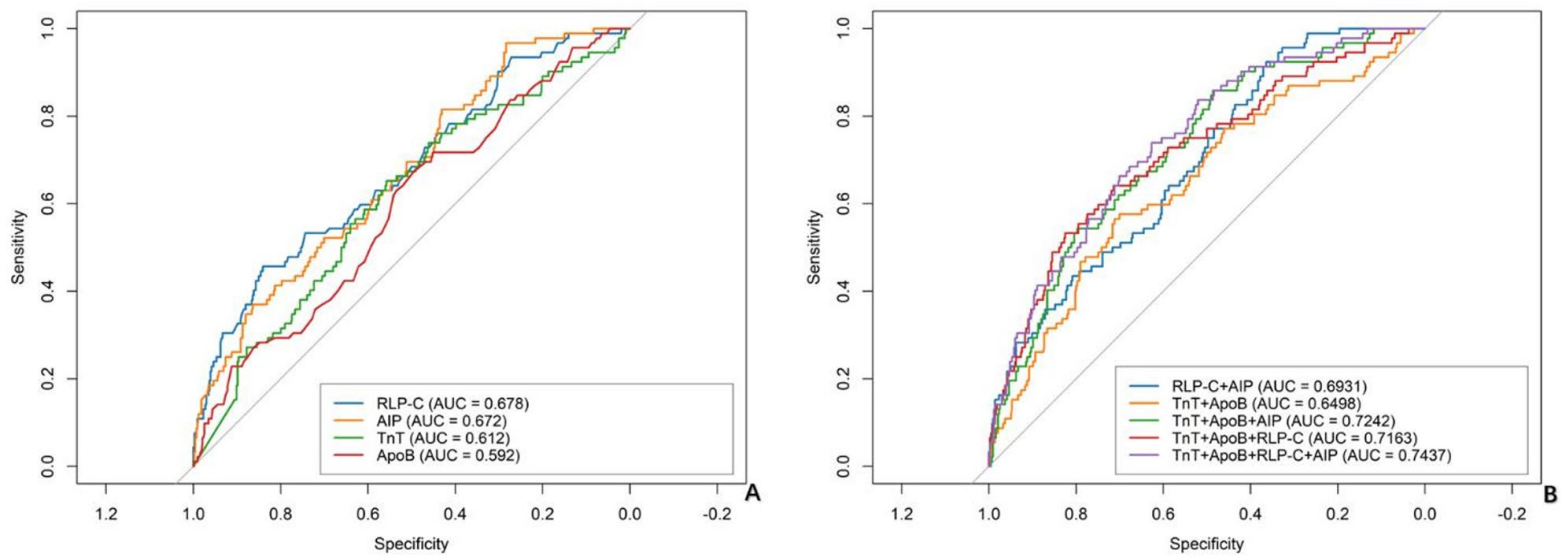
ROC for identifying MACCEs. **A** ROC curves of individual biomarkers including RLP-C, AIP, hsTnT, and ApoB. **B** ROC curves of combined biomarker models. AUC values are shown in parentheses. All models demonstrated statistically significant predictive performance (*P* < 0.05)

ROC analysis demonstrated moderate predictive value for RLP-C (AUC = 0.678, 95%CI: 0.616–0.740; *P* < 0.001) and AIP (AUC = 0.672, 95%CI: 0.613–0.731; *P* < 0.001) for in-hospital MACCEs. The combination of RLP-C and AIP improved prediction (AUC = 0.693, 95%CI: 0.638–0.749; *P* < 0.001). The four-marker model (hsTnT + ApoB + RLP-C + AIP) achieved the highest diagnostic accuracy (AUC = 0.744, 95%CI: 0.690–0.797; *P* < 0.001; sensitivity 73.9%, specificity 62.7%), significantly outperforming models based on conventional biomarkers alone (Table 4; Fig. 4).

**Table 4:** ROC curve analysis of biomarkers for predicting In-Hospital MACCEs in STEMI patients

Parameter	Cutoff	AUC	95%CI	*P*	Sensitivity(%)	Specificity(%)
AIP	0.004	0.672	0.613 ~ 0.731	< 0.001	96.7	28.3
RLP-C	0.835	0.678	0.616 ~ 0.740	< 0.001	45.7	84.1
TnT	2860.5	0.612	0.548 ~ 0.675	< 0.001	65.2	55.8
ApoB	0.895	0.592	0.528 ~ 0.655	0.005	67.4	49.5
RLP-C + AIP	—	0.693	0.638 ~ 0.749	< 0.001	92.4	36.4
TnT + ApoB	—	0.650	0.586 ~ 0.713	< 0.001	56.5	71.2
TnT + ApoB + AIP	—	0.724	0.670 ~ 0.779	< 0.001	85.9	48.6
TnT + ApoB + RLP-C	—	0.716	0.656 ~ 0.777	< 0.001	53.3	82.5
TnT + ApoB + RLP-C + AIP	—	0.744	0.690 ~ 0.797	< 0.001	73.9	62.7

The ROC curve analysis was performed to evaluate the diagnostic accuracy of individual and combined biomarkers for predicting in-hospital MACCEs in STEMI patients. AUC values and 95% confidence intervals were calculated using the DeLong method. Combined biomarker models were derived from multivariable logistic regression analyses

*MACCEs* Major adverse cardiovascular and cerebrovascular events, *hsTnT* High-sensitivity troponin T, *ApoB* Apolipoprotein B, *RLP-C* Residual lipoprotein-cholesterol, *AIP* Atherogenic index of plasma

### Dose-response relationships of RLP-C and AIP with MACCEs

**Fig. 5:**
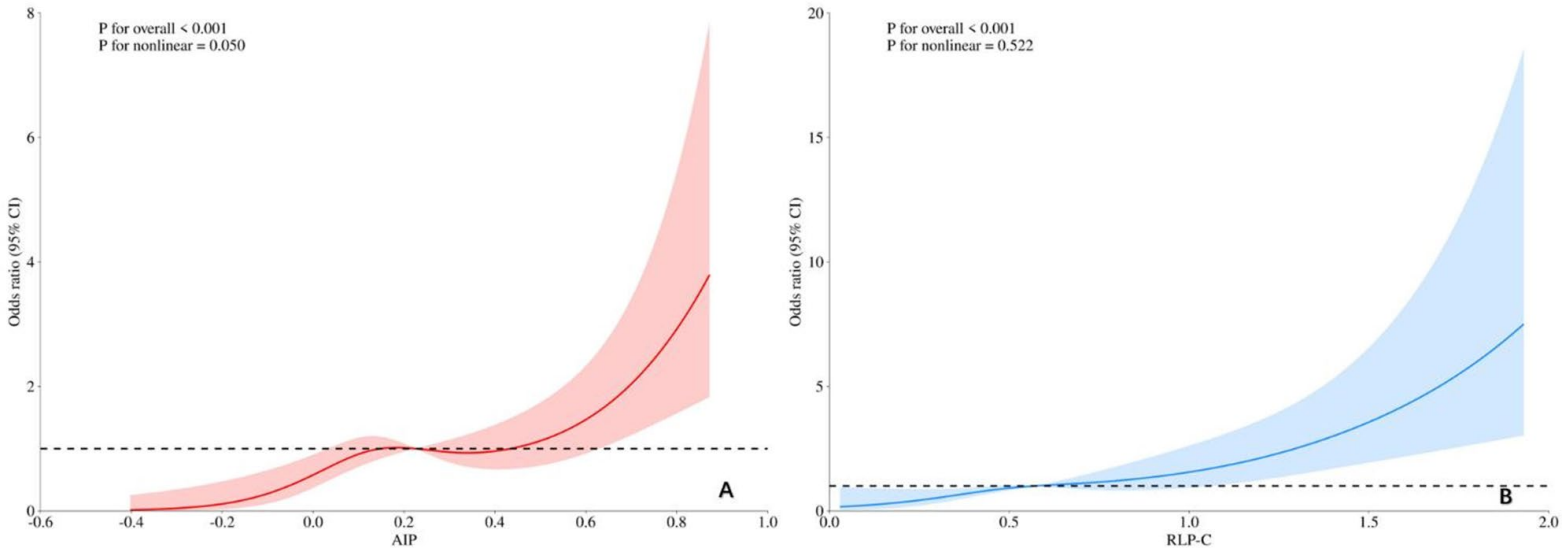
Nonlinear Dose-Response Relationships of AIP and RLP-C with In-Hospital MACCEs. **A** Association between AIP and MACCEs risk. **B** Association between RLP-C and MACCEs risk. Solid lines represent odds ratios, and shaded areas indicate 95% confidence intervals. Reference lines are set at odds ratio = 1 (horizontal)

The dose-response analysis revealed significant positive associations between both AIP and RLP-C levels and the risk of in-hospital MACCEs (P for overall < 0.001 for both). For AIP (Fig. 5A), a borderline nonlinear relationship was observed (P for nonlinearity = 0.050), suggesting that the risk increases more rapidly at higher AIP levels. In contrast, RLP-C (Fig. 5B) demonstrated a linear dose-response relationship with MACCEs (P for nonlinearity = 0.522), indicating that the risk increases proportionally with increasing RLP-C levels.

## Discussion

STEMI represents a severe manifestation of coronary artery disease (CAD), characterized by coronary atherosclerosis and thrombosis. Even after emergency PCI, patients with STEMI who received PCI remain at significant risk for in-hospital MACCEs, which adversely impact both short- and long-term prognosis and quality of life. While dyslipidemia is a well-established risk factor for coronary heart disease [17], few studies have specifically investigated the relationship between novel lipid indices and MACCEs in this patient population following PCI. Our study demonstrates that RLP-C and AIP, both individually and in combination, effectively predict in-hospital MACCEs in patients with STEMI who received PCI. This finding provides clinicians with additional tools for identifying high-risk patients and may inform more personalized therapeutic approaches.

The results showed that patients with STEMI who received PCI and developed MACCEs had significantly higher RLP-C and AIP values than those without MACCEs, suggesting both indices may predict in-hospital MACCEs after PCI. Multivariate logistic regression analysis confirmed RLP-C and AIP as independent predictors of MACCEs during STEMI hospitalization.

This study also supports previous findings that hsTnT and apolipoprotein B ApoB are independent risk factors for patients with STEMI who received PCI. Prasad et al. [18] demonstrated that 37% of patients undergoing elective PCI had elevated preoperative hsTnT levels, which may be attributed to poorer cardiovascular and metabolic risk profiles [19], more extensive CAD [20] or clinically occult complex atherosclerotic plaques [21]. The AMORIS study [22] found that among 175,553 apparently healthy individuals, ApoB demonstrated superior predictive accuracy for CVD risk compared to traditional lipid measurements including LDL-C and non-HDL-C. Saeed et al. [23] demonstrated that ApoB has prognostic significance for patients following PCI for STEMI. Correlation analysis revealed minimal associations between RLP-C, AIP and myocardial injury markers (hsTnT and CK-MB), as well as with Killip class. This weak correlation indicates that these lipid indices reflect pathophysiological processes independent of myocardial necrosis and hemodynamic status. This independence explains their persistent significance as predictors of MACCEs in multivariate analysis despite adjustment for conventional risk markers, highlighting their potential clinical utility as novel biomarkers for risk stratification in STEMI patients who received PCI.

RLP-C, a triglyceride-rich lipoprotein remnant derived from the metabolism of very-low-density lipoproteins and chylomicron (CM) particles, exhibits potent atherogenic properties through multiple mechanisms. First, RLP-C accelerates endothelial senescence by enhancing oxidative stress and impairs endothelial function by reducing nitric oxide bioavailability [24]. Second, RLP-C’s atherogenicity relates to its structural characteristics, which influence its residence time in the arterial intima, susceptibility to oxidation, permeability through the arterial wall, and ability to promote foam cell formation and plaque progression [25]. Third, RLP-C upregulates plasminogen activator inhibitor-1 (PAI-1), enhancing platelet aggregation and increasing thrombotic risk [26]. Fourth, lysophosphatidylcholine, abundant in chylomicron remnants, affects coronary endothelial function, regulates vascular smooth muscle cell early growth response factor 1 (Egr-1), and stimulates monocyte chemotactic protein-1 expression [27]. These pathophysiological mechanisms provide a biological basis for our observed association between elevated RLP-C and increased MACCEs risk. Additionally, elevated RLP-C levels correlate with ischemic stroke risk, especially large-artery atherosclerotic stroke [28]. However, according to the literature, there is no clear target value for RLP-C levels, and given its superiority as a predictor compared to other lipids, it is critical to assess RLP-C to determine the need for additional lipid-intensive therapy [29], implying that future expanded studies are required to assess the exact target value of RLP-C and to provide clinicians with a useful range of reference values to evaluate patients’ prognoses.

AIP is considered a novel indicator reflecting lipid metabolism status [30], with substantial evidence demonstrating its ability to predict atherosclerosis and cardiovascular events. In a meta-analysis, Rabiee et al. [31] concluded that AIP is a potential predictive marker for adverse cardiovascular events in patients with CAD and that higher AIP is related to an increased risk of MACE, cardiovascular death, MI, hemodialysis, and the absence of the re-flow phenomenon. Fu et al. [32] discovered that patients with Type 2 Diabetes Mellitus (T2DM) and a high AIP had a greater frequency of Major Adverse Cardiovascular Events (MACE), and AIP was an important prognostic factor for determining the risk of cardiovascular events in this population. Additionally, AIP has demonstrated predictive power for stroke in longitudinal studies, including the China Health and Retirement Longitudinal Survey (CHARLS) involving 8,727 participants aged 45 years or older (mean follow-up 8.72 years), which reported that stroke risk progressively increased with each quartile of baseline AIP level [33]. Liu et al. [34] evaluated the study and discovered that cumulative AIP had some predictive power for stroke patients, especially in the elderly. Interestingly, AIP values were calculated using laboratory tests performed at the time of the patient’s hospital admission, and variations in AIP were not observed over time. Liu et al. [34] further confirmed these findings, demonstrating that AIP had predictive value for stroke outcomes, especially among elderly patients.

Notably, the VIF for AIP was 6.440, indicating moderate multicollinearity (VIF 5–10), which is expected given its mathematical derivation as the logarithm of the TG/HDL-C ratio. Despite this, AIP remained a significant independent predictor (*P* = 0.047) in the multivariable model, whereas its individual components (TG and HDL-C) lost statistical significance (*P* = 0.236 and *P* = 0.317, respectively). This suggests that AIP captures integrated pathophysiological information beyond its components alone. Our finding aligns with the concept of atherogenic dyslipidemia as a complex metabolic state rather than an isolated lipid abnormality. While collinearity should be acknowledged, it nonetheless underscores the clinical value of AIP as a composite biomarker. Future studies in larger, independent cohorts are warranted to establish its role in risk stratification.

RLP-C and AIP may also be influenced by lifestyle factors. Dietary patterns can modulate lipid metabolism and potentially affect these biomarker levels [35]. Similarly, physical activity is known to impact lipid profiles, including remnant lipoprotein metabolism. Studies have shown that exercise interventions can reduce RLP-C levels and improve overall lipid metabolism, particularly in weight management contexts [36]. In our study of patients with STEMI who received PCI, we acknowledge that the observed associations between RLP-C, AIP and in-hospital MACCEs may be influenced by underlying lifestyle factors not directly measured in this retrospective analysis.

Our ROC analysis demonstrated that RLP-C and AIP individually exhibited moderate predictive value for in-hospital MACCEs, comparable to established biomarkers. More importantly, their combination with conventional risk markers (hsTnT and ApoB) significantly improved the model’s predictive performance. The integration of RLP-C and AIP could therefore represent a promising improvement over approaches relying solely on myocardial injury and conventional lipids. This finding has potential clinical implications: while current risk stratification for STEMI patients focuses predominantly on infarct size and hemodynamic status, our data suggest that comprehensive lipid profiling might help identify high-risk patients who could benefit from intensified secondary prevention despite successful PCI. The additive predictive value of RLP-C and AIP indicates that they capture distinct pathophysiological aspects of atherothrombosis not fully reflected by conventional biomarkers.

## Conclusion

In-hospital MACCEs following emergency PCI in STEMI patients significantly impact clinical outcomes. Our study demonstrates that RLP-C and AIP independently predict in-hospital MACCEs in patients with STEMI who received PCI, with their combination providing superior risk stratification compared to conventional markers. This approach allows clinicians to identify high-risk patients early and implement targeted preventive strategies, potentially improving outcomes. However, a prospective and larger multicenter study is warranted to confirm the predictive ability of RLP-C and AIP combination in patients with STEMI who received PCI.

## Supplementary Information

Supplementary Material 1.

## Data Availability

The datasets used and/or analysed during the current study are available from the corresponding author on reasonable request (xzdefengpan@xzhmu.edu.cn).
